# Supplementary material for: Network of interactions between the tumor necrosis factor superfamily members and small S100 proteins
Source: PLoS One. 2026 Sep 29;21(9):e0341400. doi: 10.1371/journal.pone.0341400 (PMC13623127; doi:10.1371/journal.pone.0341400)
Supplement: Supplementary file 1 [file pone.0341400.s002.docx]

**Supplementary materials**

Network of Interactions between the Tumor Necrosis Factor Superfamily Members and Small S100 Proteins

Victoria A. Rastrygina ^1^, Evgenia I. Deryusheva ^1^, Alexey S. Kazakov ^1^, Andrey S. Sokolov ^1^, Maria E. Permyakova ^1^, Ekaterina A. Litus ^1^, Vladimir N. Uversky ^2,*^, Eugene A. Permyakov ^1^, Sergei E. Permyakov ^1,*^

**^1^** Institute for Biological Instrumentation of the Russian Academy of Sciences, Institutskaya str., 7, Pushchino, Moscow Region 142290, Russia; certusfides@gmail.com (V.A.R.); janed1986@ya.ru (E.I.D.); fenixfly@yandex.ru (A.S.K.); 212sok@gmail.com (A.S.S.); mperm1977@gmail.com (M.E.P.); ealitus@gmail.com (E.A.L.); epermyak@yandex.ru (E.A.P.); permyakov.s@gmail.com (S.E.P.)

**^2^** Department of Molecular Medicine and USF Health Byrd Alzheimer’s Research Institute, Morsani College of Medicine, University of South Florida, Tampa, FL 33612, USA; vuversky@usf.edu (V.N.U.)

***** Correspondence: vuversky@usf.edu (V.N.U.); permyakov.s@gmail.com (S.E.P.); Tel.: +7-(977)-838-54-40 (S.E.P.); Fax: +7-(4967)-33-05-22 (S.E.P.)

**Figure S1.** SPR spectroscopy data on kinetics of interaction between the TNFSF members immobilized on SPR sensor by amine coupling and S100 proteins or human CaM/PV/HSA (1 μM) at 25°C, in the absence/presence of Ca^2+^: panel **A**, CD70; **B**, CD30L; **C**, 4-1BBL; **D**, TWEAK; **E**, APRIL; **F**, LIGHT; **G**, VEGI; **H**, AITRL. Buffer conditions: 10 mM HEPES-NaOH, 150 mM NaCl, 0.05% TWEEN 20, pH 7.4 with 1 mM CaCl_2_ (‘+ Ca^2+^’) or 5 mM EDTA (‘+ EDTA’). The association phase is marked by the vertical dashed lines. The SPR sensograms are colored as follows: S100A1 (*cyan*), S100A2 (*orange*), S100A4 (*blue*), S100A5 (*fuchsia*), S100A6 (*navy*), S100A16 (*gray*), S100B (*violet*), S100P (*green*), CaM (*salmon*) in the absence of Ca^2+^; S100A2 (*dark orange*), S100A5 (*magenta*), S100A6 (*dark blue*), S100P (*bright green*), S100P Δ42–47 (*teal*), S100P F89A (*lime*), CaM (*red*), PV (*brown*), HSA (*dark violet*) in the presence of Ca^2+^.

**Figure S2**. The structural models of complexes between AITRL (*green*) and Ca^2+^-loaded S100 dimers (*grey* and *cyan*) built using ClusPro docking server *[1]*. The Ca^2+^-binding loops and the contact residues are shown in *yellow* and *orange*, respectively.

**Figure S3.** The structural models of complexes between Ca^2+^-loaded S100A2 dimers (*grey* and *cyan*) and TNFSF members (*green*) built using ClusPro docking server *[1]*. The Ca^2+^-binding loops and the contact residues are shown in *yellow* and *orange*, respectively**.**

**Table S1**. The samples of soluble forms of TNFSF representatives studied in this work and refs. *[2, 3]* (marked as ^†^) for affinity to non-fused S100 proteins and their characteristics. The amino acid residues of tags are underlined.

| *Abbreviation* | *Full name* | *UniProt ID* | *Manufacturer, cat. #* | *Expression system* | *Amino acid sequence* | *Molecular mass, kDa* | *Calculated pI [4]* | *ε_280nm_, M^-1^cm^-1^ [5]* |
| --- | --- | --- | --- | --- | --- | --- | --- | --- |
| *TNFSF members specific to S100* | | | | | | | | |
| TNF^†^ | TNF ligand superfamily member 2 | P01375 | ProSpec, CYT-223 | *E. coli* | MVRSSSRTPS DKPVAHVVAN PQAEGQLQWL NRRANALLAN GVELRDNQLV VPSEGLYLIY SQVLFKGQGC PSTHVLLTHT ISRIAVSYQT KVNLLSAIKS PCQRETPEGA EAKPWYEPIY LGGVFQLEKG DRLSAEINRP DYLDFAESGQ VYFGIIAL | 17.5 | 6.8 | 21,555 |
| CD70 | TNF ligand superfamily member 7 | P32970 | ProSpec, CYT-953 | Insect cells (Sf9) | ADPQRFAQAQ QQLPLESLGW DVAELQLNHT GPQQDPRLYW QGGPALGRSF LHGPELDKGQ LRIHRDGIYM VHIQVTLAIC SSTTASRHHP TTLAVGICSP ASRSISLLRL SFHQGCTIAS QRLTPLARGD TLCTNLTGTL LPSRNTDETF FGVQWVRPHH HHHH | 18.2 | 8.5 | 19,730 |
| CD30L | TNF ligand superfamily member 8 | P32971 | ProSpec, CYT-824 | *E. coli* | MGSSHHHHHH SSGLVPRGSH MGSQRTDSIP NSPDNVPLKG GNCSEDLLCI LKRAPFKKSW AYLQVAKHLN KTKLSWNKDG ILHGVRYQDG NLVIQFPGLY FIICQLQFLV QCPNNSVDLK LELLINKHIK KQALVTVCES GMQTKHVYQN LSQFLLDYLQ VNTTISVNVD TFQYIDTSTF PLENVLSIFL YSNSD | 22.1 | 8.6 | 21,680 |
| 4-1BBL | TNF ligand superfamily member 9 | P41273 | ProSpec, CYT-149 | *E. coli* | REGPELSPDD PAGLLDLRQG MFAQLVAQNV LLIDGPLSWY SDPGLAGVSL TGGLSYKEDT KELVVAKAGV YYVFFQLELR RVVAGEGSGS VSLALHLQPL RSAAGAAALA LTVDLPPASS EARNSAFGFQ GRLLHLSAGQ RLGVHLHTEA RARHAWQLTQ GATVLGLFRV TPEIPAGLPS PRSE | 19.4 | 6.2 | 16,960 |
| TRAIL^†^ | Tumor necrosis factor ligand superfamily member 10 | P50591 | PeproTech, 310-04 | *E. coli* | MRERGPQRVA AHITGTRGRS NTLSSPNSKN EKALGRKINS WESSRSGHSF LSNLHLRNGE LVIHEKGFYY IYSQTYFRFQ EEIKENTKND KQMVQYIYKY TSYPDPILLM KSARNSCWSK DAEYGLYSIY QGGIFELKEN DRIFVSVTNE HLIDMDHEAS FFGAFLVG | 19.5 | 8.9 | 27,390 |
| TWEAK | TNF ligand superfamily member 12 | O43508 | PeproTech, 310-06 | *E. coli* | MKGRKTRARR AIAAHYEVHP RPGQDGAQAG VDGTVSGWEE ARINSSSPLR YNRQIGEFIV TRAGLYYLYC QVHFDEGKAV YLKLDLLVDG VLALRCLEEF SATAASSLGP QLRLCQVSGL LALRPGSSLR IRTLPWAHLK AAPFLTYFGL FQVH | 17.10 | 9.6 | 21,555 |
| APRIL | TNF ligand superfamily member 13 | O75888 | PeproTech, 310-10C | Insect cells | AVLTQKQKKQ HSVLHLVPIN ATSKDDSDVT EVMWQPALRR GRGLQAQGYG VRIQDAGVYL LYSQVLFQDV TFTMGQVVSR EGQGRQETLF RCIRSMPSHP DRAYNSCYSA GVFHLHQGDI LSVIIPRARA KLNLSPHGTF LGFVKL | 16.4 | 9.8 | 13,075 |
| LIGHT | TNF ligand superfamily member 14 | O43557 | PeproTech, 310-09B | Insect cells | RLGEMVTRLP DGPAGSWEQL IQERRSHEVN PAAHLTGANS SLTGSGGPLL WETQLGLAFL RGLSYHDGAL VVTKAGYYYI YSKVQLGGVG CPLGLASTIT HGLYKRTPRY PEELELLVSQ QSPCGRATSS SRVWWDSSFL GGVVHLEAGE KVVVRVLDER LVRLRDGTRS YFGAFMV | 19.4 | 8.6 | 34,045 |
| VEGI | TNF ligand superfamily member 15 | O95150-2 | ProSpec, CYT-517 | *E. coli* | MQLTKGRLHF SHPLSHTKHI SPFVTDAPLR ADGDKPRAHL TVVRQTPTQH FKNQFPALHW EHELGLAFTK NRMNYTNKFL LIPESGDYFI YSQVTFRGMT SECSEIRQAG RPNKPDSITV VITKVTDSYP EPTQLLMGTK SVCEVGSNWF QPIYLGAMFS LQEGDKLMVN VSDISLVDYT KEDKTFFGAF LL | 21.9 | 7.8 | 20,065 |
| AITRL | TNF ligand superfamily member 18 | Q9UNG2 | ProSpec, CYT-076 | *E. coli* | MQLETAKEPC MAKFGPLPSK WQMASSEPPC VNKVSDWKLE ILQNGLYLIY GQVAPNANYN DVAPFEVRLY KNKDMIQTLT NKSKIQNVGG TYELHVGDTI DLIFNSEHQV LKNNTYWGII LLANPQFIS | 14.6 | 6.1 | 25,565 |
| *TNFSF members non-specific to S100* | | | | | | | | |
| TNF-β^†^ | TNF ligand superfamily member 1 | P01374 | BioVision, 4345 | *E. coli* | LPGVGLTPSA AQTARQHPKM HLAHSTLKPA AHLIGDPSKQ NSLLWRANTD RAFLQDGFSL SNNSLLVPTS GIYFVYSQVV FSGKAYSPKA TSSPLYLAHE VQLFSSQYPF HVPLLSSQKM VYPGLQEPWL HSMYHGAAFQ LTQGDQLSTH TDGIPHLVLS PSTVFFGAFA L | 18.7 | 8.9 | 21,430 |
| OX40L | TNF ligand superfamily member 4 | P23510 | PeproTech, 310-28 | Insect cells | QVSHRYPRIQ SIKVQFTEYK KEKGFILTSQ KEDEIMKVQN NSVIINCDGF YLISLKGYFS QEVNISLHYQ KDEEPLFQLK KVRSVNSLMV ASLTYKDKVY LNVTTDNTSL DDFHVNGGEL ILIHQNPGEF CVL | 15.4 | 6.5 | 10,555 |
| CD40L | TNF ligand superfamily member 5 | P29965 | PeproTech, 310-02 | *E. coli* | MQKGDQNPQI AAHVISEASS KTTSVLQWAE KGYYTMSNNL VTLENGKQLT VKRQGLYYIY AQVTFCSNRE ASSQAPFIAS LWLKSPGRFE RILLRAANTH SSAKPCGQQS IHLGGVFELQ PGASVFVNVT DPSQVSHGTG FTSFGLLKL | 16.3 | 9.4 | 18,575 |
| FasL | TNF ligand superfamily member 6 | P48023 | ProSpec, CYT-051 | HEK293 | HHHHHHPSPP PEKKELRKVA HLTGKSNSRS MPLEWEDTYG IVLLSGVKYK KGGLVINETG LYFVYSKVYF RGQSCNNLPL SHKVYMRNSK YPQDLVMMEG KMMSYCTTGQ MWARSSYLGA VFNLTSADHL YVNVSELSLV NFEESQTFFG LYKL | 17.7 | 9.1 | 27,515 |
| RANKL | TNF ligand superfamily member 11 | O14788 | PeproTech, 310-01 | *E. coli* | MEKAMVDGSW LDLAKRSKLE AQPFAHLTIN ATDIPSGSHK VSLSSWYHDR GWAKISNMTF SNGKLIVNQD GFYYLYANIC FRHHETSGDL ATEYLQLMVY VTKTSIKIPS SHTLMKGGST KYWSGNSEFH FYSINVGGFF KLRSGEEISI EVSNPSLLDP DQDATYFGAF KVRDID | 19.9 | 6.4 | 35,410 |
| BAFF | TNF ligand superfamily member 13B | Q9Y275 | PeproTech, 310-13 | *E. coli* | AVQGPEETVT QDCLQLIADS ETPTIQKGSY TFVPWLLSFK RGSALEEKEN KILVKETGYF FIYGQVLYTD KTYAMGHLIQ RKKVHVFGDE LSLVTLFRCI QNMPETLPNN SCYSAGIAKL EEGDELQLAI PRENAQISLD GDVTFFGALK LL | 17.0 | 4.9 | 14,565 |

**Table S2**. The structures of the Ca^2+^-loaded S100 dimers and TNFSF members used for structural modelling of their complexes using ClusPro docking server [1].

| *Protein* | *PDB entry* | *Chains* | *Method* | *Comments* |
| --- | --- | --- | --- | --- |
| *S100 proteins* | | | | |
| S100A1 | 2LP3 | A, B | NMR |  |
| S100A2 | 4DUQ | A, B | X-ray |  |
| S100A4 | 2Q91 | A, B | X-ray |  |
| S100A5 | 2KAY | A, B | NMR |  |
| S100A6 | 1K9K | A, B | X-ray |  |
| S100A11 | n.a. | - | AlphaFold 3 | model with 4 Ca^2+^ ions |
| S100A12 | 1E8A | A, B | X-ray |  |
| S100A13 | 1YUT | A, B | NMR |  |
| S100A16 | 2L51 | A, B | NMR |  |
| S100B | 1MQ1 | A, B | NMR | complex with TRTKIDWNKILS |
| S100P | 2MJW | B, D | NMR | complex with RAGE |
| *TNFSF members* | | | | |
| TNF | 3WD5 | A | X-ray | complex with Fab fragment of Adalimumab;  residues 77-233 are used for docking  (THD domain: residues 88-233)* |
| CD70 | 7KX0 | A | X-ray | complex with CD27;  residues 39-193 are used for docking  (THD domain: residues 56-191)* |
| CD30L | n.a. | - | AlphaFold 3 | residues 83-234 are used for docking  (THD domain: residues 98-225)* |
| 4-1BBL | 6MGE | A | X-ray | residues 58-254 are used for docking  (THD domain: residues 91-240)* |
| TRAIL | 1D4V | B | X-ray | complex with DR5; residues 119-281 are used for docking  (THD domain: residues 122-280)* |
| TWEAK | u.r. | - | AlphaFold 3 | residues 97-249 are used for docking  (THD domain: residues 107-248)* |
| APRIL | u.r. | - | AlphaFold 3 | residues 105-250 are used for docking  (THD domain: residues 116-250)* |
| LIGHT | u.r. | - | AlphaFold 3 | residues 83-240 are used for docking  (THD domain: residues 94-240)* |
| VEGI | 2O0O | A | X-ray | residues 72-251 are used for docking  (THD domain: residues 95-251)* |
| AITRL | 2Q1M | A | X-ray | residues 52-177 are used for docking  (THD domain: residues 47-170)* |

n.a., data not available;

u.r., unresolved residues are present in PDB [6] files;

*, according to UniProt **[7]**

**Table S3**. The contact residues of the complexes between Ca^2+^-loaded S100 dimers and TNFSF members predicted using ClusPro docking server [1]. Residue numbering is according to the PDB [6] entries (Table S2). See also Figure 7.

| *TNFSF member* | *S100 protein* | *Contact residues of S100 proteins* | | *Contact residues of TNFSF members* |
| --- | --- | --- | --- | --- |
| TNF | S100A11 | Chain A | Chain B | T2,P3,S4,H10,Q16,R27,A28,N29,L52,Y54,Y110,Y114,E141,Y146,I150,L152 |
|  |  | I4,E15,F95,A98 | M1,I4,E15,F95 |  |
|  | S100A12 | Chain A | Chain B | R1,T2,P3,S4,K6,A9,H10,R27,A28,N29,L31,Y54,Y114,E141,S142,G143,Y146,I149,I150 |
|  |  | H85,Y86,H87 | E8,N12,H15,V19,R20,H23,F24,D25 |  |
|  | S100A13 | Chain A | Chain B | T2,P3,S4,K6,H10,R27,N29,L31,L52,Y54,Q56,K93,Y114,S142,G143,Y146,I150,L152 |
|  |  | M1,A2,A3,E4,P5,L6,T7,E8,E10,E11 | R25,Q26,N36,E40,T43,Q44,Q45,H48 |  |
| CD70 | S100A2 | Chain A | Chain B | R91,Q103,R104,L105,R139 |
|  |  | F44,D50,G53,S60,E63,V76,L80,V83,M84,D87,F88 | S60,E63,D70,Q72 |  |
| CD30L | S100A1 | Chain A | Chain B | S1,E2,D3,L4,C6,I7,K9,R10,A11,P12,F13,F58,I60,F111,L113,L139,F146,Y148 |
|  |  | E9,N13,F89,N92 | E9,N13,F89,N92 |  |
|  | S100A2 | Chain A | Chain B | S1,E2,I7,R10,P12,F13,F58,K88,F111,L113,Y115,F146,Y148,S149 |
|  |  | Q74,D89,F90 | - |  |
|  | S100A4 | Chain A | Chain B | S1,E2,I7,K9,R10,P12,F13,K14,F58,F111,F146,Y148 |
|  |  | - | F44,M84,F88 |  |
|  | S100A5 | Chain A | Chain B | S1,I7,R10,P12,F13,F58,I60,F111,F146,Y148,S149 |
|  |  | M1,E2 | L44,F86 |  |
|  | S100A6 | Chain A | Chain B | S1,E2,I7,R10,P12,F13,F58,F111,L113,Y115,F146,Y148 |
|  |  | I83,Y84,E86,K89 | - |  |
|  | S100B | Chain A | Chain B | S1,E2,I7,R10,A11,P12,F13,K14,F58,I60,K88,F111,L113,Y115,F146,Y148,S149 |
|  |  | E45,K48,E91 | - |  |
|  | S100P | Chain B | Chain D | S1,E2,L4,I7,R10,P12,L56,F58,I60,F111,L113,Y115,F146,Y148 |
|  |  | C85,Y88,F89,A92,L94 | M1,E5,S16,Q26 |  |
| 4-1BBL | S100A6 | Chain A | Chain B | G2,M3,F4,Q6,G26,L27,G29,S31,L32,V52,Y54,F56,F111,G113,R114,L115,F150,R151,V152,T153 |
|  |  | Q6 | - |  |
| TRAIL | S100P | Chain B | Chain D | H7,F45,F63,Y65,Y67,Q69,Y125,I129,F156,F160,V162,G163 |
|  |  | P42,G43,Y88,F89,A92,G93 | M1,E5 |  |
| TWEAK | S100A5 | Chain A | Chain B | R4,K5,T6,R7,A8,R9,R10,A11,I12,I43,N44,R62,Y67,F151,Q152,V153,H154 |
|  |  | M1 | L44,E46,A82,Y83,D85,F86,E89 |  |
|  | S100A12 | Chain A | Chain B | R9,R10,A11,H15,I43,N44,R62,Y67,Y69,L120,P143,Y147,F151,Q152,V153,H154 |
|  |  | N12,H15,V19,R20,K21,H23,F24,D25 | Y86,H87,T88 |  |
| APRIL | S100A1 | Chain A | Chain B | K6,K8,Q10,H11,V13,H15,L38,R40,L60,Y62,Q64,Y108,A110,F140,V144,L146 |
|  |  | G1,E3,T6,E9,N13,N92 | E3,E9,N13,F89 |  |
|  | S100A2 | Chain A | Chain B | K8,H11,V13,H15,R40,L60,Y62,Q64,R94,Y108,V112,L146 |
|  |  | F44,K48,G53,S60,E63,M84,D87,F88 | K56,S60,E63,Q72 |  |
|  | S100A5 | Chain A | Chain B | H15,P36,A37,L38,R40,Y62,Y108,P136,F140 |
|  |  | M1 | L44,E46,K48,D54,K57,M81,A82,Y83,D85,F86,E89,D90 |  |
|  | S100B | Chain A | Chain B | K8,K9,H11,H15,R40,Y62,Q64,I93,R94,S106,Y108,S109,A110,V112,F113,H114,H137,F140,L146 |
|  |  | E58,T59,L60,N62,Q71,M79,A83,E86 | T59,L60,N62,D63,D65,Q71,M79,A83,C84,E86,F87,F88,E91 |  |
| LIGHT | S100A2 | Chain A | Chain B | R5,R6,H8,E9,N11,H15,G37,L38,F40,R42,Y60,Y62,Y152,F156,M157,V158 |
|  |  | S60,E63,V76,L80 | S43,F44,K56,E63,Q72,V76,L80,M84,D87,F88 |  |
|  | S100A5 | Chain A | Chain B | C63,L65,T100,S102,R104,R132,R141 |
|  |  |  | L44,E46,A82,Y83,D85,F86,E89 |  |
|  | S100B | Chain A | Chain B | Q3,E4,R5,R6,H8,E9,N11,H15,G37,F40,R42,Y58,Y60,Y62,T149,Y152,F156,M157,V158 |
|  |  | K48,T59,L60,N62,Q71,M79,A83,C84,E86,F88 | K48,T59,L60,N62,Q71,M79,A83,C84 |  |
| VEGI | S100P | Chain B | Chain D | A6,H7,L34,F36,F57,Y59,Y115,F151 |
|  |  | Y88,F89,A92,L94 | M1,E5 |  |
| AITRL | S100A1 | Chain A | Chain B | P1,C2,K5,P10,S11,M15,S17,S18,E19,P20,N24,K25,Q35,Y42,L113,A115,N116 |
|  |  | N86,W90,E91,S93 | H16,Y26 |  |
|  | S100A2 | Chain A | Chain B | P1,M3,K5,L40,Y42,G43,N79,T83,E85,Y108,I112,L114 |
|  |  | F44,D87,F88 | - |  |
|  | S100A4 | Chain A | Chain B | K5,L40,Y42,D66,I68,N79,T83,Y84,E85,H87,I112,L114 |
|  |  | F44,K47,R48,N60,M84,F88,K95,Q96 | - |  |
|  | S100A5 | Chain A | Chain B | Y42,T83,N116 |
|  |  | - | E46,K48,S51,M78,A82,D85 |  |
|  | S100A6 | Chain A | Chain B | M3,L40,Y42,G43,I68,Q69,T70,N79,G81,G82,T83,Y84,Y108,I112 |
|  |  | R54,D58,Y83,E85,A86,K88 | A1 |  |
|  | S100A16 | Chain A | Chain B | P1,M3,K5,L40,Y42,N79,T83,I112,L114,N116 |
|  |  | H47,S50,D51,L92,Q99,S102 | S1,D2,C3,Y4 |  |
|  | S100B | Chain A | Chain B | M3,K5,Q14,M15,L40,Y42,Q44,N79,T83,N105,N106,Y108,I112,L114,N116 |
|  |  | F88,E91 | - |  |
|  | S100P | Chain B | Chain D | P1,M3,K5,Q14,L40,Y42,N79,I112 |
|  |  | M1,E5,P42,G43,Y88,F89,A92 | M1,T2,E5,Y88,F89,A92 |  |

**References**

1.	Desta IT, Porter KA, Xia B, Kozakov D, Vajda S. Performance and Its Limits in Rigid Body Protein-Protein Docking. Structure. 2020;28(9):1071-81 e3. Epub 2020/07/11. doi: 10.1016/j.str.2020.06.006. PubMed PMID: 32649857; PubMed Central PMCID: PMC7484347.

2.	Rastrygina VA, Kazakov AS, Fadeev RS, Meshcheriakova EI, Deryusheva EI, Sokolov AS, et al. Soluble form of tumor necrosis factor-related apoptosis-inducing ligand interacts with S100P protein. Int J Biol Macromol. 2025:143667. Epub 20250501. doi: 10.1016/j.ijbiomac.2025.143667. PubMed PMID: 40318722.

3.	Kazakov AS, Zemskova MY, Rystsov GK, Vologzhannikova AA, Deryusheva EI, Rastrygina VA, et al. Specific S100 Proteins Bind Tumor Necrosis Factor and Inhibit Its Activity. Int J Mol Sci. 2022;23(24):15956. Epub 20221215. doi: 10.3390/ijms232415956. PubMed PMID: 36555597; PubMed Central PMCID: PMCPMC9783754.

4.	Wilkins MR, Gasteiger E, Bairoch A, Sanchez JC, Williams KL, Appel RD, et al. Protein identification and analysis tools in the ExPASy server. Methods Mol Biol. 1999;112:531-52. doi: 10.1385/1-59259-584-7:531. PubMed PMID: 10027275.

5.	Pace CN, Vajdos F, Fee L, Grimsley G, Gray T. How to measure and predict the molar absorption coefficient of a protein. Protein Sci. 1995;4(11):2411-23. PubMed PMID: 46.

6.	Berman HM, Westbrook J, Feng Z, Gilliland G, Bhat TN, Weissig H, et al. The Protein Data Bank. Nucleic Acids Res. 2000;28(1):235-42. PubMed PMID: 12.

7.	UniProt C. UniProt: the Universal Protein Knowledgebase in 2023. Nucleic Acids Res. 2023;51(D1):D523-D31. doi: 10.1093/nar/gkac1052. PubMed PMID: 36408920; PubMed Central PMCID: PMCPMC9825514.
