# Supplementary material for: Network of interactions between the tumor necrosis factor superfamily members and small S100 proteins
Source: PLoS One. 2026 Sep 29;21(9):e0341400. doi: 10.1371/journal.pone.0341400 (PMC13623127; doi:10.1371/journal.pone.0341400)
Supplement: Peer review history

# Peer review: Decision Letter 0

**DOI:** 10.1371/journal.pone.0341400.r001

**Authors:** Abhinava Kumar Mishra

26 Apr 2026

Dear Dr. Uversky,

Thank you for submitting your manuscript to PLOS ONE. After careful consideration, we feel that it has merit but does not fully meet PLOS ONE’s publication criteria as it currently stands. Therefore, we invite you to submit a revised version of the manuscript that addresses the points raised during the review process.

==============================

The manuscript explores an interesting and relevant topic with clear clinical importance. However, the current version requires substantial revision to improve clarity, strengthen the overall structure, and better define its contribution to the field. A major revision is needed before the manuscript can be considered further. Please refer to reviewer's comment for detailed issues.

==============================

Please submit your revised manuscript by Jun 10 2026 11:59PM. If you will need more time than this to complete your revisions, please reply to this message or contact the journal office at plosone@plos.org. When you're ready to submit your revision, log on to https://www.editorialmanager.com/pone/ and select the 'Submissions Needing Revision' folder to locate your manuscript file.

- A letter that responds to each point raised by the academic editor and reviewer(s). You should upload this letter as a separate file labeled 'Response to Reviewers'.
- A marked-up copy of your manuscript that highlights changes made to the original version. You should upload this as a separate file labeled 'Revised Manuscript with Track Changes'.
- An unmarked version of your revised paper without tracked changes. You should upload this as a separate file labeled 'Manuscript'.

If you would like to make changes to your financial disclosure, please include your updated statement in your cover letter. Guidelines for resubmitting your figure files are available below the reviewer comments at the end of this letter.

If applicable, we recommend that you deposit your laboratory protocols in protocols.io to enhance the reproducibility of your results. Protocols.io assigns your protocol its own identifier (DOI) so that it can be cited independently in the future. For instructions see: https://journals.plos.org/plosone/s/submission-guidelines#loc-laboratory-protocols. Additionally, PLOS ONE offers an option for publishing peer-reviewed Lab Protocol articles, which describe protocols hosted on protocols.io. Read more information on sharing protocols at https://plos.org/protocols?utm_medium=editorial-email&utm_source=authorletters&utm_campaign=protocols.

As the corresponding author, your ORCID iD is verified in the submission system and will appear in the published article. PLOS supports the use of ORCID, and we encourage all coauthors to register for an ORCID iD and use it as well. Please encourage your coauthors to verify their ORCID iD within the submission system before final acceptance, as unverified ORCID iDs will not appear in the published article. *Only*  the individual author can complete the verification step; PLOS staff *cannot*  verify ORCID iDs on behalf of authors.

We look forward to receiving your revised manuscript.

Kind regards,

Abhinava Kumar Mishra, PhD

Academic Editor

PLOS One

Journal Requirements:

When submitting your revision, we need you to address these additional requirements.

1. Please ensure that your manuscript meets PLOS ONE's style requirements, including those for file naming. The PLOS ONE style templates can be found at

https://journals.plos.org/plosone/s/file?id=wjVg/PLOSOne_formatting_sample_main_body.pdf and

https://journals.plos.org/plosone/s/file?id=ba62/PLOSOne_formatting_sample_title_authors_affiliations.pdf.

2. Please note that PLOS One has specific guidelines on code sharing for submissions in which author-generated code underpins the findings in the manuscript. In these cases, we expect all author-generated code to be made available without restrictions upon publication of the work. Please review our guidelines at https://journals.plos.org/plosone/s/materials-and-software-sharing#loc-sharing-code and ensure that your code is shared in a way that follows best practice and facilitates reproducibility and reuse.

3. Thank you for stating the following financial disclosure:

“This research was funded by Russian Science Foundation, grant № 25-14-00223 to S.E.P. “

Please state what role the funders took in the study.  If the funders had no role, please state: "The funders had no role in study design, data collection and analysis, decision to publish, or preparation of the manuscript."

If this statement is not correct you must amend it as needed.

Please include this amended Role of Funder statement in your cover letter; we will change the online submission form on your behalf.

4. Please be informed that funding information should not appear in the Acknowledgments section or other areas of your manuscript. We will only publish funding information present in the Funding Statement section of the online submission form. Please remove any funding-related text from the manuscript.

5. If the reviewer comments include a recommendation to cite specific previously published works, please review and evaluate these publications to determine whether they are relevant and should be cited. There is no requirement to cite these works unless the editor has indicated otherwise.

[Note: HTML markup is below. Please do not edit.]

Reviewers' comments:

Reviewer's Responses to Questions

**Comments to the Author**

1. Is the manuscript technically sound, and do the data support the conclusions?

Reviewer #1: Yes

Reviewer #2: Partly

Reviewer #3: Yes

**********

2. Has the statistical analysis been performed appropriately and rigorously?

Reviewer #1: Yes

Reviewer #2: N/A

Reviewer #3: Yes

**********

3. Have the authors made all data underlying the findings in their manuscript fully available?

The PLOS Data policy

Reviewer #1: Yes

Reviewer #2: Yes

Reviewer #3: Yes

**********

4. Is the manuscript presented in an intelligible fashion and written in standard English?

Reviewer #1: Yes

Reviewer #2: Yes

Reviewer #3: Yes

**********

Reviewer #1: The manuscript characterizes interactions between TNFSF members and S100 proteins using SPR, structural modeling, and limited mutagenesis. The dataset is extensive and provides insight into potential crosstalk between these protein families. Overall, the study is interesting and well performed. The following suggestions may help further strengthen the manuscript:

1. While the biochemical interaction data are convincing, the functional relevance of these interactions remains to be further clarified. The manuscript suggests that S100 proteins may modulate TNFSF signaling; therefore, it would be valuable to include selected functional validation experiments, for example assessing whether S100 proteins influence TNFSF-mediated responses in cultured cells (e.g., inflammatory or signaling readouts). Even limited proof-of-principle experiments would strengthen the physiological significance of the findings.

2. Further clarification or discussion of the physiological or pathological contexts in which these interactions are most relevant would improve interpretation.

3. A brief justification of the SPR fitting models (e.g., heterogeneous ligand versus one-site/two-state models) would be helpful for readers.

4. The limitations of rigid-body docking could be more explicitly discussed when interpreting residue-level predictions.

Reviewer #2: In this manuscript, Rastrygina et al provide a systematic analysis of the interactions between 13 distinct members of the Tumor Necrosis Factor superfamily (TNFSF) and the 21 distinct S100 proteins present in humans, using SPR methodology. Unravelling 27 specific, calcium-dependent TNFSF:S100 interactions, including many new interactions that have not yet been reported in the literature, they further analyze the similarities and specificities of these different interactions, using docking to structurally model the resulting complexes and evaluation of disorder propensity in both partners to draw some general conclusions on the binding mode between TNFSF members and S100 proteins.

This is a very interesting piece of work that brings another layer of complexity in the interacting network of both S100 and TNFSF proteins, suggesting the first ones may act as regulator of the second ones’ activity, especially during pathological inflammation and associated disorders, which may have important consequences for the therapeutic targeting of TNFSF-dependent signalling with anti-inflammatory aim. The SPR data and their interpretation are quite robust; on the other hand, the structural modelling of the interactions and the conclusions drawn from this part deserve, in my opinion, further investigation and comprehensive analysis. I would therefore like to see the following questions/concerns addressed before the manuscript can be considered suitable for publication in PLOS One:

1) The oligomeric state of both TNFSF and S100 proteins, as well as the stoichiometry of the interactions, are not discussed at all in the manuscript. From the proposed docking model, it is taken that a monomeric TNFSF interacts with a dimeric S100 protein, while TNFSF members are known to form trimers even in solution. This is a very important point to address in this study. Do S100 proteins bind to the trimeric, active form or do they readily bind to a monomeric TNFSF (and may this possibly be compatible with binding of several S100 dimers to one TNFSF trimer)? Therefore, the real stoichiometry of the TNFSF:S100 complexes should be investigated with another methodology, preferably in solution to avoid bias in the formation of the full complex due to immobilization of one of the partner.

2) There are now many excellent AI-based tools to structurally model protein complexes. Have the authors tried to model the TNFSF:S100 complexes with such tools? What does AlphaFold modelling suggest as compared to the docking models proposed by the authors? Having corroboration of the proposed interaction modes by some other prediction tools would give more strength to these models, especially since the experimental validation provided on the S100P example is not fully satisfying.

3) It is overall difficult to assess the diversity of interaction modes between S100 and TNFSF proteins as only 2 docking models are displayed in Figure 7. A few more models should be represented, possibly highlighting the diversity of binding interfaces. In particular, the authors insist on the fact that the same region in S100s (predominantly the hinge region) is involved in the contact with all TNFSFs. However the overall binding modes displayed in Fig. 7A and 7B seem rather different. The authors should comment on that and better illustrate the resemblances and differences in overall TNFSF:S100 complexation.

4) In line with the previous point: for one given TNFSF, do all S100 binders interact the same way? This should be illustrated by a figure overlaying the different TNFSF:S100 complexes for one given TNFSF, for example CD30L or AITRL that bind to quite a few S100.

5) Except for the hinge region, the regions of contact with TNFSF within S100 proteins are mostly proposed to be in the N- and C-termini regions, that are unstructured and highly flexible by nature, and that also display most divergence in terms of sequence conservation. This is rather surprising and does not give much strength to the docking models, especially since the interactions are not illustrated at the atomic level in the figures. In particular, the initiator methionine is proposed as a contact residue for several S100, although it may not even be present in the mature protein in vivo. This questions the relevance of the proposed interfaces, knowing that the sole experimental validation performed on S100P mutants does not fully agree with the docking models. The authors should comment on these discrepancies and provide further experimental evidence of the proposed interfaces, by performing mutagenesis analysis on other S100 proteins, notably the 2 for which complexes are displayed in Figure 7.

6) The authors demonstrated the calcium-dependency of the interactions measured by SPR. Do the proposed docking models provide a rationale for this Ca-dependency? The authors should overlay the structures of apo-S100s on these complexes to corroborate that the S100 conformational state in apo form is not favourable to the proposed interaction mode.

Minor comments:

7) Introduction, line 39: Crohn’s disease and psoriasis are not autoimmune diseases stricto sensu. Please rephrase to ‘one of the most successful targets for treatment of autoimmune and inflammatory diseases’.

Reviewer #3: Comments to the Author:

In the present manuscript, Victoria A. Rastrygina et al. identify a novel cooperation implicating the S100 calcium-binding proteins and the members of the TNF superfamily. Authors have carried out surface plasmon resonance spectroscopy and purified recombinant proteins to establish an interaction network. This study expands the previously published observation from this author’s group characterizing the interplay of S100A11, S100A12, S100A13/TNF, and S100P/TRAIL. The present manuscript describes the original study, where the selectivity of interactions between soluble forms of 13 TNF members and 21 S100 proteins is explored with subsequent quantification of the cross-reactivity of S100 proteins to prone target recognition. These data are original, establishing an interaction network between two distinguishable classes of proteins.

1. The study presents the results of original research - Yes

2. Results reported have not been published elsewhere - Yes

3. Experiments, statistics, and other analyses are performed to a high technical standard and are described in sufficient detail - Yes

4. Conclusions are presented in an appropriate fashion and are supported by the data - Yes, however, several issues need to be addressed by the authors:

While these interactions as such are very interesting, the data otherwise remains short of explaining the complexes of S100/TNFSF physiological significance at least in some pathological conditions. Particular interest considering the complexes that are “orphans” (prone to selective target recognition), namely S100A12, S100A13, and S100A16. For example, S100A16 interacts with AITRL, which is highly expressed in endothelial cells, affecting T cell activity. Is S100A16 also expressed in endothelial cells? The latter presumes the extracellular existence of both factors in the same niche and the possible functional outcome of their complex formation.

The authors state that: “Since the interactions of S100 proteins with TNFSF representatives characterized to date inhibit activity of the latter [17,18], it is likely that the identified TNFSF-S100 interactions serve as a mechanism for suppression of extracellular signaling in the case of cell damage” (lines 380-381); and ….”the TNFSF-S100 interactions are attractive for targeted pharmacological interventions modulating these interactions. Furthermore, S100 protein levels may need to be considered upon use and development of the drugs targeting TNFSF member signaling to ensure their maximum efficacy” (lines 383-385). Would the authors be more specific about what they presumed and how it is possible when uniform binding sites are not identified and domains involved in protein interaction are so diverse within each group of cytokines and S100 proteins? Does this statement assume the increasing extracellular level of S100 proteins depletes TNFSF member signaling? Because the elevated level of S100 proteins is often associated with different pathologies, the authors should be more specific on what they talk about.

5. The article is presented in an intelligible fashion and is written in standard English –Yes, with except of misspelling: “…with the cytokines are somewht more ordered (line 337), meaning somewhat? and, “Similarly, most 100 proteins (with the exception to S100A11, S100A13…” (line 353), meaning S100 proteins?

6. The research meets all applicable standards for the ethics of experimentation and research integrity - Yes

7. The article adheres to appropriate reporting guidelines and community standards for data availability – Yes.

**********

what does this mean?). If published, this will include your full peer review and any attached files.

If you choose “no”, your identity will remain anonymous but your review may still be made public.

**Do you want your identity to be public for this peer review?**  For information about this choice, including consent withdrawal, please see our Privacy Policy

Reviewer #1: **Yes:**  Qing Lin

Reviewer #2: **Yes:**  Laure Yatime

Reviewer #3: No

**********

[NOTE: If reviewer comments were submitted as an attachment file, they will be attached to this email and accessible via the submission site. Please log into your account, locate the manuscript record, and check for the action link "View Attachments". If this link does not appear, there are no attachment files.]

To ensure your figures meet our technical requirements, please review our figure guidelines: https://journals.plos.org/plosone/s/figures

You may also use PLOS’s free figure tool, NAAS, to help you prepare publication quality figures: https://journals.plos.org/plosone/s/figures#loc-tools-for-figure-preparation.

NAAS will assess whether your figures meet our technical requirements by comparing each figure against our figure specifications.

# Author comment: Author response to Decision Letter 1

**DOI:** 10.1371/journal.pone.0341400.r002

29 Jun 2026

PONE-D-26-00579

Network of Interactions between the Tumor Necrosis Factor Superfamily Members and Small S100 Proteins

PLOS One

Dear Dr. Uversky,

Thank you for submitting your manuscript to PLOS ONE. After careful consideration, we feel that it has merit but does not fully meet PLOS ONE’s publication criteria as it currently stands. Therefore, we invite you to submit a revised version of the manuscript that addresses the points raised during the review process.

==============================

The manuscript explores an interesting and relevant topic with clear clinical importance. However, the current version requires substantial revision to improve clarity, strengthen the overall structure, and better define its contribution to the field. A major revision is needed before the manuscript can be considered further. Please refer to reviewer's comment for detailed issues.

==============================

Please submit your revised manuscript by Jun 10 2026 11:59PM. If you will need more time than this to complete your revisions, please reply to this message or contact the journal office at plosone@plos.org. When you're ready to submit your revision, log on to https://www.editorialmanager.com/pone/ and select the 'Submissions Needing Revision' folder to locate your manuscript file.

Please include the following items when submitting your revised manuscript:

• A letter that responds to each point raised by the academic editor and reviewer(s). You should upload this letter as a separate file labeled 'Response to Reviewers'.

• A marked-up copy of your manuscript that highlights changes made to the original version. You should upload this as a separate file labeled 'Revised Manuscript with Track Changes'.

• An unmarked version of your revised paper without tracked changes. You should upload this as a separate file labeled 'Manuscript'.

If you would like to make changes to your financial disclosure, please include your updated statement in your cover letter. Guidelines for resubmitting your figure files are available below the reviewer comments at the end of this letter.

If applicable, we recommend that you deposit your laboratory protocols in protocols.io to enhance the reproducibility of your results. Protocols.io assigns your protocol its own identifier (DOI) so that it can be cited independently in the future. For instructions see: https://journals.plos.org/plosone/s/submission-guidelines#loc-laboratory-protocols. Additionally, PLOS ONE offers an option for publishing peer-reviewed Lab Protocol articles, which describe protocols hosted on protocols.io. Read more information on sharing protocols at https://plos.org/protocols?utm_medium=editorial-email&utm_source=authorletters&utm_campaign=protocols.

As the corresponding author, your ORCID iD is verified in the submission system and will appear in the published article. PLOS supports the use of ORCID, and we encourage all coauthors to register for an ORCID iD and use it as well. Please encourage your coauthors to verify their ORCID iD within the submission system before final acceptance, as unverified ORCID iDs will not appear in the published article. Only the individual author can complete the verification step; PLOS staff cannot verify ORCID iDs on behalf of authors.

We look forward to receiving your revised manuscript.

Kind regards,

Abhinava Kumar Mishra, PhD

Academic Editor

PLOS One

Journal Requirements:

When submitting your revision, we need you to address these additional requirements.

1. Please ensure that your manuscript meets PLOS ONE's style requirements, including those for file naming. The PLOS ONE style templates can be found at

https://journals.plos.org/plosone/s/file?id=wjVg/PLOSOne_formatting_sample_main_body.pdf and https://journals.plos.org/plosone/s/file?id=ba62/PLOSOne_formatting_sample_title_authors_affiliations.pdf.

2. Please note that PLOS One has specific guidelines on code sharing for submissions in which author-generated code underpins the findings in the manuscript. In these cases, we expect all author-generated code to be made available without restrictions upon publication of the work. Please review our guidelines at https://journals.plos.org/plosone/s/materials-and-software-sharing#loc-sharing-code and ensure that your code is shared in a way that follows best practice and facilitates reproducibility and reuse.

3. Thank you for stating the following financial disclosure:

“This research was funded by Russian Science Foundation, grant № 25-14-00223 to S.E.P. “

Please state what role the funders took in the study. If the funders had no role, please state: "The funders had no role in study design, data collection and analysis, decision to publish, or preparation of the manuscript." If this statement is not correct you must amend it as needed.

Please include this amended Role of Funder statement in your cover letter; we will change the online submission form on your behalf.

4. Please be informed that funding information should not appear in the Acknowledgments section or other areas of your manuscript. We will only publish funding information present in the Funding Statement section of the online submission form. Please remove any funding-related text from the manuscript.

5. If the reviewer comments include a recommendation to cite specific previously published works, please review and evaluate these publications to determine whether they are relevant and should be cited. There is no requirement to cite these works unless the editor has indicated otherwise.

Reviewers' comments:

Comments to the Author

1. Is the manuscript technically sound, and do the data support the conclusions?

The manuscript must describe a technically sound piece of scientific research with data that supports the conclusions. Experiments must have been conducted rigorously, with appropriate controls, replication, and sample sizes. The conclusions must be drawn appropriately based on the data presented.

Reviewer #1: Yes

Reviewer #2: Partly

Reviewer #3: Yes

________________________________________

2. Has the statistical analysis been performed appropriately and rigorously?

Reviewer #1: Yes

Reviewer #2: N/A

Reviewer #3: Yes

________________________________________

3. Have the authors made all data underlying the findings in their manuscript fully available?

The PLOS Data policy requires authors to make all data underlying the findings described in their manuscript fully available without restriction, with rare exception (please refer to the Data Availability Statement in the manuscript PDF file). The data should be provided as part of the manuscript or its supporting information, or deposited to a public repository. For example, in addition to summary statistics, the data points behind means, medians and variance measures should be available. If there are restrictions on publicly sharing data—e.g. participant privacy or use of data from a third party—those must be specified.

Reviewer #1: Yes

Reviewer #2: Yes

Reviewer #3: Yes

________________________________________

4. Is the manuscript presented in an intelligible fashion and written in standard English?

PLOS ONE does not copyedit accepted manuscripts, so the language in submitted articles must be clear, correct, and unambiguous. Any typographical or grammatical errors should be corrected at revision, so please note any specific errors here.

Reviewer #1: Yes

Reviewer #2: Yes

Reviewer #3: Yes

________________________________________

5. Review Comments to the Author

Please use the space provided to explain your answers to the questions above. You may also include additional comments for the author, including concerns about dual publication, research ethics, or publication ethics. (Please upload your review as an attachment if it exceeds 20,000 characters)

Reviewer #1

The manuscript characterizes interactions between TNFSF members and S100 proteins using SPR, structural modeling, and limited mutagenesis. The dataset is extensive and provides insight into potential crosstalk between these protein families. Overall, the study is interesting and well performed. The following suggestions may help further strengthen the manuscript:

1. While the biochemical interaction data are convincing, the functional relevance of these interactions remains to be further clarified. The manuscript suggests that S100 proteins may modulate TNFSF signaling; therefore, it would be valuable to include selected functional validation experiments, for example assessing whether S100 proteins influence TNFSF-mediated responses in cultured cells (e.g., inflammatory or signaling readouts). Even limited proof-of-principle experiments would strengthen the physiological significance of the findings.

RESPONSE: The potential functional significance of some interactions between S100 proteins and TNFSF members has already been demonstrated in the cell-based assays of soluble TNF (PMID: 36555597) and TRAIL (PMID: 40318722). These studies are mentioned in the Abstract, Introduction, Results and discussion (section 3.1) and Conclusions. We believe that each such interaction deserves a separate detailed study of its potential functional significance. An attempt to extend the present study with additional functional data would dilute the key message of this manuscript.

2. Further clarification or discussion of the physiological or pathological contexts in which these interactions are most relevant would improve interpretation.

RESPONSE: We intentionally avoided excessive discussion of these aspects, as the both protein families are involved in a wide range of (patho)physiological processes. In the Conclusions section, we provide only qualitative assessment of potential significance of these interactions. A more detailed assessment would need the analysis exemplified by our previous papers (PMID: 36555597, 40318722) for each of the novel interactions, which is excessive for the purposes of this work. We believe that further speculations will degrade quality of the manuscript.

3. A brief justification of the SPR fitting models (e.g., heterogeneous ligand versus one-site/two-state models) would be helpful for readers.

RESPONSE: We have added the following explanation to section 2.2: “The simplest fitting model ensuring the lowest χ2 value was used”.

4. The limitations of rigid-body docking could be more explicitly discussed when interpreting residue-level predictions.

RESPONSE: We have added the following explanations of limitations of the rigid body approximation to section 3.2: “In fact, the rigid body modelling may underestimate number of the contact residues, since it does not account for possible conformational changes during the interactions. Nevertheless, this approach is valuable for assessment of spatial arrangement of the interacting molecules”.

Reviewer #2

In this manuscript, Rastrygina et al provide a systematic analysis of the interactions between 13 distinct members of the Tumor Necrosis Factor superfamily (TNFSF) and the 21 distinct S100 proteins present in humans, using SPR methodology. Unravelling 27 specific, calcium-dependent TNFSF:S100 interactions, including many new interactions that have not yet been reported in the literature, they further analyze the similarities and specificities of these different interactions, using docking to structurally model the resulting complexes and evaluation of disorder propensity in both partners to draw some general conclusions on the binding mode between TNFSF members and S100 proteins.

This is a very interesting piece of work that brings another layer of complexity in the interacting network of both S100 and TNFSF proteins, suggesting the first ones may act as regulator of the second ones’ activity, especially during pathological inflammation and associated disorders, which may have important consequences for the therapeutic targeting of TNFSF-dependent signalling with anti-inflammatory aim. The SPR data and their interpretation are quite robust; on the other hand, the structural modelling of the interactions and the conclusions drawn from this part deserve, in my opinion, further investigation and comprehensive analysis. I would therefore like to see the following questions/concerns addressed before the manuscript can be considered suitable for publication in PLOS One:

1) The oligomeric state of both TNFSF and S100 proteins, as well as the stoichiometry of the interactions, are not discussed at all in the manuscript. From the proposed docking model, it is taken that a monomeric TNFSF interacts with a dimeric S100 protein, while TNFSF members are known to form trimers even in solution. This is a very important point to address in this study. Do S100 proteins bind to the trimeric, active form or do they readily bind to a monomeric TNFSF (and may this possibly be compatible with binding of several S100 dimers to one TNFSF trimer)? Therefore, the real stoichiometry of the TNFSF:S100 complexes should be investigated with another methodology, preferably in solution to avoid bias in the formation of the full complex due to immobilization of one of the partner.

RESPONSE: Indeed, S100 proteins tend to dimerize, whereas TNFSF members are prone to trimerization. This fact greatly complicates rigorous characterization of their interactions. Our previous study of stoichiometry of the interaction between sTRAIL and S100P using intrinsic fluorescence and chemical crosslinking revealed a complicated picture (PMID: 40318722), namely, existence of multiple calcium-dependent equilibria between several complexes differing in the molar ratio of S100P to sTRAIL (at least 1:3, 1:1, and 2:1, respectively). Clearly, rigorous characterization of such interrelated chemical equilibria represents a fundamental challenge. Therefore, here we aimed to simplify this system as much as possible while maintaining its functional significance. Monomerization of TNFSF members is one of such approaches. Since the prototypical member of TNFSF, TNF, is known to exist in a monomeric form at physiologically relevant picomolar levels (PMID: 1622406), we believe that monomeric forms of TNFSF members are of physiological significance. The conditions of our SPR experiments with TNFSF members used as a ligand favor their monomerization. Therefore, the molecular docking was performed for monomeric forms of TNFSF members.

We have added the following clarifications on this issue to section 3.1: “Note that conditions of the SPR experiments favor monomerization of the TNFSF members, whereas formation of active signaling complexes with their cognate receptors involves trimerization of the ligand [2]. However, monomeric forms of TNFSF members are presumably of physiological significance, given that the prototypical TNFSF member, TNF, is known to exist in a monomeric form at physiologically relevant picomolar levels [18,68]. The previous study of stoichiometry of the interaction between soluble TRAIL and S100P using intrinsic fluorescence and chemical crosslinking revealed a complicated picture, namely, presence of multiple equilibria between several complexes differing in the molar ratio of S100P to sTRAIL (1:3, 1:1, and 2:1, respectively [17]). Nevertheless, the resulting estimates of the apparent equilibrium dissociation constants of the complexes were in qualitative agreement with the SPR estimates”.

2) There are now many excellent AI-based tools to structurally model protein complexes. Have the authors tried to model the TNFSF:S100 complexes with such tools? What does AlphaFold modelling suggest as compared to the docking models proposed by the authors? Having corroboration of the proposed interaction modes by some other prediction tools would give more strength to these models, especially since the experimental validation provided on the S100P example is not fully satisfying.

RESPONSE: Yes, we confirmed some of our modelling results using AlphaFold3, which gave somewhat different results at the level of individual amino acid residues, but relative spatial orientation of the interacting molecules was well preserved. Since the interactions studied are strictly calcium-dependent, we believe that the use of experimental tertiary structures of calcium-loaded S100 proteins for structural modelling in this particular case is more appropriate. Furthermore, it was important to compare the modelling results with our previous data on S100 interactions with various cytokines, obtained using the same methodology (PMIDs: 42190582, 35053268, 33322098,35216109, 36233301, 36555597, 37759746, 40318722, 38484433).

3) It is overall difficult to assess the diversity of interaction modes between S100 and TNFSF proteins as only 2 docking models are displayed in Figure 7. A few more models should be represented, possibly highlighting the diversity of binding interfaces. In particular, the authors insist on the fact that the same region in S100s (predominantly the hinge region) is involved in the contact with all TNFSFs. However the overall binding modes displayed in Fig. 7A and 7B seem rather different. The authors should comment on that and better illustrate the resemblances and differences in overall TNFSF:S100 complexation.

RESPONSE: To improve presentation of the modelling results, we introduced the following changes into the manuscript:

- moved from supplementary materials to the main text the scheme of the predicted contact residues of the TNFSF-S100 complexes mapped onto the aligned amino acid sequences of the binding partners;

- added the figures illustrating the models of AITRL complexes with eight S100 proteins (Figure S2) and the models of S100A2 complexes with five TNFSF members (Figure S3);

- made the appropriate changes to the main text and supplementary materials.

In addition, we have added the following text to this section: “Meanwhile, the modeling of S100A2 complexes with five TNFSF members shows that the latter can bind to S100A2 from the opposite sides of its dimer (Figure S3). In fact, the rigid body modelling may underestimate number of the contact residues, since it does not account for possible conformational changes during the interactions. Nevertheless, this approach is valuable for assessment of spatial arrangement of the interacting molecules”.

4) In line with the previous point: for one given TNFSF, do all S100 binders interact the same way? This should be illustrated by a figure overlaying the different TNFSF:S100 complexes for one given TNFSF, for example CD30L or AITRL that bind to quite a few S100.

RESPONSE: We have included the figure illustrating models of AITRL complexes with eight S100 proteins, in which position of AITRL molecule is fixed (Figure S2). This figure shows that the predicted position of the S100-binding site of AITRL is quite conserved, as indicated in the text (“..despite noticeable variation in positions of the contact residues in the aligned sequences of the both interaction partners, locations of their binding sites are well conserved”).

5) Except for the hinge region, the regions of contact with TNFSF within S100 proteins are mostly proposed to be in the N- and C-termini regions, that are unstructured and highly flexible by nature, and that also display most divergence in terms of sequence conservation. This is rather surprising and does not give much strength to the docking models, especially since the interactions are not illustrated at the atomic level in the figures. In particular, the initiator methionine is proposed as a contact residue for several S100, although it may not even be present in the mature protein in vivo. This questions the relevance of the proposed interfaces, knowing that the sole experimental validation performed on S100P mutants does not fully agree with the docking models. The authors should comment on these discrepancies and provide further experimental evidence of the proposed interfaces, by performing mutagenesis analysis on other S100 proteins, notably the 2 for which complexes are displayed in Figure 7.

RESPONSE:

- Since the rigid body modelling used here relies upon known tertiary structures of the interacting partners, we had to consider the methionine in the first position if it was present in the original structure.

- To improve presentation of the modelling results, we added the figures illustrating the models of AITRL complexes with eight S100 proteins (Figure S2) and the models of S100A2 complexes with five TNFSF members (Figure S3).

- Indeed, some S100 proteins tend to be disordered (PMID: 21528128), but their known tertiary structures show that their amino acid residues are mostly resolved. Furthermore, S100 proteins have very similar tertiary structures, despite large diversity of their primary structures. Therefore, we see no contradiction in the idea that S100 proteins may share a relatively conserved cytokine-binding site, which may depend on the specific S100 protein.

- The rigid body modelling does not account for structural flexibility of the interacting partners, so it is unable to provide fully reliable structures of the complexes. Therefore, the partial discrepancy between our experimental data for the S100P mutants and the theoretical predictions is fairly expected. Nevertheless, the rigid body approximation is valuable for assessment of spatial arrangement of the interacting molecules. We have added the following clarification to section 3.2: “In fact, the rigid body modelling may underestimate number of the contact residues, since it does not account for possible conformational changes during the interactions. Nevertheless, this approach is valuable for assessment of spatial arrangement of the interacting molecules”.

- Given the aforementioned limitations of the rigid body modeling, further experiments with mutant forms of other S100 proteins are expected to yield a picture very similar to that observed for the S100P mutants. Thus, the additional experiments will significantly delay the publication without adequate yield.

6) The authors demonstrated the calcium-dependency of the interactions measured by SPR. Do the proposed docking models provide a rationale for this Ca-dependency? The authors should overlay the structures of apo-S100s on these complexes to corroborate that the S100 conformational state in apo form is not favourable to the proposed interaction mode.

RESPONSE: Indeed, overlay of the known tertiary structures of S100P in apo- and Ca2+-loaded states (PMIDs: 12507480, 15213440) shows that the protein regions most affected by calcium binding are the ‘hinge’ and C-terminal fragment, which are both predicted to participate in recognition of the TNFSF members (Figure 7A). This fact may explain the strong Ca2+ dependence of these S100P interactions. We have added these considerations to section 3.2: “Importantly, namely these regions of S100P (‘hinge’ and C-terminal fragment) have previously been shown to be most affected by Ca2+ binding (PMIDs: 12507480, 15213440), which may explain the strong Ca2+ dependence of these S100P interactions”.

Minor comments:

7) Introduction, line 39: Crohn’s disease and psoriasis are not autoimmune diseases stricto sensu. Please rephrase to ‘one of the most successful targets for treatment of autoimmune and inflammatory diseases’.

RESPONSE: Corrected

Reviewer #3:

In the present manuscript, Victoria A. Rastrygina et al. identify a novel cooperation implicating the S100 calcium-binding proteins and the members of the TNF superfamily. Authors have carried out surface plasmon resonance spectroscopy and purified recombinant proteins to establish an interaction network. This study expands the previously published observation from this author’s group characterizing the interplay of S100A11, S100A12, S100A13/TNF, and S100P/TRAIL. The present manuscript describes the original study, where the selectivity of interactions between soluble forms of 13 TNF members and 21 S100 proteins is explored with subsequent quantification of the cross-reactivity of S100 proteins to prone target recognition. These data are original, establishing an interaction network between two distinguishable classes of proteins.

1. The study presents the results of original research - Yes

2. Results reported have not been published elsewhere – Yes

3. Experiments, statistics, and other analyses are performed to a high technical standard and are described in sufficient detail – Yes

4. Conclusions are presented in an appropriate fashion and are supported by the data - Yes, however, several issues need to be addressed by the authors:

While these interactions as such are very interesting, the data otherwise remains short of explaining the complexes of S100/TNFSF physiological significance at least in some pathological conditions. Particular interest considering the complexes that are “orphans” (prone to selective target recognition), namely S100A12, S100A13, and S100A16. For example, S100A16 interacts with AITRL, which is highly expressed in endothelial cells, affecting T cell activity. Is S100A16 also expressed in endothelial cells? The latter presumes the extracellular existence of both factors in the same niche and the possible functional outcome of their complex formation.

RESPONSE: S100A16 is enriched in endothelial cells of kidney and pituitary gland, and serves as a prognostic marker in kidney renal clear cell carcinoma and some other cancers (liver hepatocellular carcinoma, lung adenocarcinoma, pancreatic adenocarcinoma) – see https://www.proteinatlas.org/ENSG00000188643-S100A16. Thus, the possibility suggested by the reviewer is quite probable. Nevertheless, we intentionally avoid excessive discussion of such aspects, since the both protein families are involved in a wide range of (patho)physiological processes. In the Conclusions chapter, we provide only qualitative assessment of potential significance of these interactions. A more detailed assessment would need the analysis exemplified by our previous papers (PMID: 36555597, 40318722) for each of the novel interactions, which is excessive for the purposes of this work. We believe that further speculations will degrade quality of the manuscript.

The authors state that: “Since the interactions of S100 proteins with TNFSF representatives characterized to date inhibit activity of the latter [17,18], it is likely that the identified TNFSF-S100 interactions serve as a mechanism for suppression of extracellular signaling in the case of cell damage” (lines 380-381); and ….”the TNFSF-S100 interactions are attractive for targeted pharmacological interventions modulating these interactions. Furthermore, S100 protein levels may need to be considered upon use and development of the drugs targeting TNFSF member signaling to ensure their maximum efficacy” (lines 383-385). Would the authors be more specific about what they presumed and how it is possible when uniform binding sites are not identified and domains involved in protein interaction are so diverse within each group of cytokines and S100 proteins? Does this statement assume the increasing extracellular level of S100 proteins depletes TNFSF member signaling? Because the elevated level of S100 proteins is often associated with different pathologies, the authors should be more specific on what they talk about.

RESPONSE: We have added the following clarifications to the text: “The elevated levels of S100 proteins in most cancers [38-41] may suppress activity of individual TNFSF members, which may interfere with the therapies aimed at activation of their signaling (for instance, treatment of soft tissue sarcoma and multiple myeloma by recombinant TNF and circularly permuted TRAIL, respectively [11]). Thus, targeted inhibition of such negative interactions could be a promising therapeutic option. Therefore, S100 protein levels may need to be considered upon use and development of the drugs targeting TNFSF member signaling to ensure their maximum efficacy”.

5. The article is presented in an intelligible fashion and is written in standard English –Yes, with except of misspelling: “…with the cytokines are somewht more ordered (line 337), meaning somewhat? and, “Similarly, most 100 proteins (with the exception to S100A11, S100A13…” (line 353), meaning S100 proteins?

RESPONSE: Corrected

6. The research meets all applicable standards for the ethics of experimentation and research integrity - Yes

7. The article adheres to appropriate reporting guidelines and community standards for data availability – Yes.

________________________________________

6. PLOS authors have the option to publish the peer review history of their article (what does this mean?). If published, this will include your full peer review and any attached files.

If you choose “no”, your identity will remain anonymous but your review may still be made public.

Do you want your identity to be public for this peer review? For information about this choice, including consent withdrawal, please see our Privacy Policy.

Reviewer #1: Yes: Qing Lin

Reviewer #2: Yes: Laure Yatime

Reviewer #3: No

________________________________________

[NOTE: If reviewer comments were submitted as an attachment file, they will be attached to this email and accessible via the submission site. Please log into your account, locate the manuscript record, and check for the action link "View Attachments". If this link does not appear, there are no attachment files.]

Attachment Submitted filename: Response to reviewers.docx

# Peer review: Decision Letter 1

**DOI:** 10.1371/journal.pone.0341400.r003

**Authors:** Andreas Hofmann

7 Sep 2026

Network of Interactions between the Tumor Necrosis Factor Superfamily Members and Small S100 Proteins

PONE-D-26-00579R1

Dear Dr. Uversky,

Based on the comments provided by the reviewers who also commented on the initial submission we’re pleased to inform you that your manuscript has been judged scientifically suitable for publication and will be formally accepted for publication once it meets all outstanding technical requirements.

Within one week, you’ll receive an e-mail detailing the required amendments. When these have been addressed, you’ll receive a formal acceptance letter and your manuscript will be scheduled for publication.

An invoice will be generated when your article is formally accepted. Please note, if your institution has a publishing partnership with PLOS and your article meets the relevant criteria, all or part of your publication costs will be covered. Please make sure your user information is up-to-date by logging into Editorial Manager at Editorial Manager® and clicking the ‘Update My Information' link at the top of the page. For questions related to billing, please contact billing support.

If your institution or institutions have a press office, please notify them about your upcoming paper to help maximize its impact. If they’ll be preparing press materials, please inform our press team as soon as possible -- no later than 48 hours after receiving the formal acceptance. Your manuscript will remain under strict press embargo until 2 pm Eastern Time on the date of publication. For more information, please contact onepress@plos.org.

Kind regards,

Andreas Hofmann

Academic Editor

PLOS One

Additional Editor Comments (optional):

Reviewers' comments:

Reviewer's Responses to Questions

**Comments to the Author**

Reviewer #1: All comments have been addressed

Reviewer #2: All comments have been addressed

Reviewer #3: All comments have been addressed

**********

2. Is the manuscript technically sound, and do the data support the conclusions?

Reviewer #1: Yes

Reviewer #2: Yes

Reviewer #3: Yes

**********

3. Has the statistical analysis been performed appropriately and rigorously?

Reviewer #1: Yes

Reviewer #2: Yes

Reviewer #3: Yes

**********

4. Have the authors made all data underlying the findings in their manuscript fully available?

The PLOS Data policy

Reviewer #1: Yes

Reviewer #2: Yes

Reviewer #3: Yes

**********

5. Is the manuscript presented in an intelligible fashion and written in standard English?

Reviewer #1: Yes

Reviewer #2: Yes

Reviewer #3: Yes

**********

Reviewer #1: The revised manuscript has improved, and the authors have addressed my main comments. Although no new functional validation experiments were added, I find the authors’ explanation reasonable given the intended scope of this work.

Reviewer #2: In this revised manuscript, Rastrygina et al have provided valuable new information that greatly helped assessing the relevance of the S100-TNFSF interactions they describe. In particular, addition of a proper justification for the use of a monomeric model for TNFSF was important and gives more strength to the modelling study. The addition of supplementary Figures S2 and S3, which highlight the various S100-TNFSF complexes for one given TNFSF and one given S100, is also a very important add-on that allows to better visualize the range of variation in the S100-TNFSF binding mode for one given protein. Although I understand there is limited space in the main text, I believe these figures may be worth having as main figures rather than in supplementary material. Overall, the authors have provided very convincing explanation on the rationale behind their modelling strategy and the general interpretation they derive from these newly discovered interactions. While adding on the physiological relevance of these complexes would clearly be interesting, it is perfectly understandable that it goes beyond the scope of this current manuscript and would deserve further investigations in follow-up studies of specific S100-TNFSF complexes. With the current improvement, I find the manuscript now suitable for publication in PLoS ONE.

Reviewer #3: All comments are answered. I believe this publication will make basis for further experimental studies showing functional importance of TNFSF members and S100 proteins interaction.

**********

what does this mean?). If published, this will include your full peer review and any attached files.

If you choose “no”, your identity will remain anonymous but your review may still be made public.

**Do you want your identity to be public for this peer review?**  For information about this choice, including consent withdrawal, please see our Privacy Policy

Reviewer #1: **Yes:**  Qing Lin

Reviewer #2: **Yes:**  Laure Yatime

Reviewer #3: **Yes:**  Marina Y. Zemskova

**********

# Editor report: Acceptance letter

**DOI:** 10.1371/journal.pone.0341400.r004

**Authors:** Andreas Hofmann

PONE-D-26-00579R1

PLOS One

Dear Dr. Uversky,

I'm pleased to inform you that your manuscript has been deemed suitable for publication in PLOS One. Congratulations! Your manuscript is now being handed over to our production team.

At this stage, our production department will prepare your paper for publication. This includes ensuring the following:

* All references, tables, and figures are properly cited

* All relevant supporting information is included in the manuscript submission,

* There are no issues that prevent the paper from being properly typeset

You will receive further instructions from the production team, including instructions on how to review your proof when it is ready. Please keep in mind that we are working through a large volume of accepted articles, so please give us a few days to review your paper and let you know the next and final steps.

Lastly, if your institution or institutions have a press office, please let them know about your upcoming paper now to help maximize its impact. If they'll be preparing press materials, please inform our press team within the next 48 hours. Your manuscript will remain under strict press embargo until 2 pm Eastern Time on the date of publication. For more information, please contact onepress@plos.org.

You will receive an invoice from PLOS for your publication fee after your manuscript has reached the completed accept phase. If you receive an email requesting payment before acceptance or for any other service, this may be a phishing scheme. Learn how to identify phishing emails and protect your accounts at https://explore.plos.org/phishing.

If we can help with anything else, please email us at customercare@plos.org.

Thank you for submitting your work to PLOS One and supporting open access.

Kind regards,

PLOS One Editorial Office Staff

on behalf of

Dr Andreas Hofmann

Academic Editor

PLOS One
